# Cognitive Variability during Middle-Age: Possible Association with Neurodegeneration and Cognitive Reserve

**DOI:** 10.3389/fnagi.2017.00188

**Published:** 2017-06-09

**Authors:** Daniel Ferreira, Alejandra Machado, Yaiza Molina, Antonieta Nieto, Rut Correia, Eric Westman, José Barroso

**Affiliations:** ^1^Division of Clinical Geriatrics–Center for Alzheimer Research, Department of Neurobiology, Care Sciences and Society, Karolinska InstitutetStockholm, Sweden; ^2^Faculty of Psychology, University of La LagunaLa Laguna, Spain; ^3^Faculty of Health Sciences, University Fernando Pessoa CanariasLas Palmas, Spain; ^4^Facultad de Educación, Universidad Diego PortalesSantiago, Chile

**Keywords:** middle-age, cognition, cognitive reserve, cortical thickness, mean diffusivity, white matter hyperintensities, subjective memory complaints, depressive symptomatology

## Abstract

**Objective:** Increased variability in cognition with age has been argued as an indication of pathological processes. Focusing on early detection of neurodegenerative disorders, we investigated variability in cognition in healthy middle-aged adults. In order to understand possible determinants of this variability, we also investigated associations with cognitive reserve, neuroimaging markers, subjective memory complaints, depressive symptomatology, and gender.

**Method:** Thirty-one 50 ± 2 years old individuals were investigated as target group and deviation was studied in comparison to a reference younger group of 30 individuals 40 ± 2 years old. Comprehensive neuropsychological and structural imaging protocols were collected. Brain regional volumes and cortical thickness were calculated with FreeSurfer, white matter hyperintensities with CASCADE, and mean diffusivity with FSL.

**Results:** Across-individuals variability showed greater dispersion in lexical access, processing speed, executive functions, and memory. Variability in global cognition correlated with, reduced cortical thickness in the right parietal-temporal-occipital association cortex, and increased mean diffusivity in the cingulum bundle and right inferior fronto-occipital fasciculus. A trend was also observed for the correlation between global cognition and hippocampal volume and female gender. All these associations were influenced by cognitive reserve. No correlations were found with subjective memory complaints, white matter hyperintensities and depressive symptomatology. Across-domains and across-tasks variability was greater in several executive components and cognitive processing speed.

**Conclusion:** Variability in cognition during middle-age is associated with neurodegeneration in the parietal–temporal–occipital association cortex and white matter tracts connecting this to the prefrontal dorsolateral cortex and the hippocampus. Moreover, this effect is influenced by cognitive reserve. Studying variability offers valuable information showing that differences do not occur in the same magnitude and direction across individuals, cognitive domains and tasks. These findings may have important implications for early detection of subtle cognitive impairment and clinical interpretation of deviation from normality.

## Introduction

Cognitive aging has been extensively studied mostly focusing on old age (Willis et al., 2010). However, factors affecting brain structure and function exert continuous and cumulative influences across the whole life-span (Sperling et al., 2011; Walhovd et al., 2014). Accumulation of aging-related pathology in the brain may reach a certain threshold, usually during middle-age, triggering different biological changes or chronic diseases that may lead to cognitive decline in old age (Cerhan et al., 1998; Lachman, 2004; Debette et al., 2011). For example, in disorders such as Alzheimer’s disease (AD), neurodegeneration is evident while individuals are still cognitively normal, 10 to 20 years prior to dementia diagnosis (Sperling et al., 2011; Villemagne et al., 2013). For this reason, research is now focusing more and more on middle-age with the hope of uncovering mechanisms that allow initiating interventions as early as possible.

Middle-age is often considered as a period of little or no cognitive change (Lachman, 2004; Willis et al., 2010). However, subtle cognitive decline has been reported in several studies (Salthouse, 2009; Zimprich and Mascherek, 2010; Gautam et al., 2011; Singh-Manoux et al., 2012; Ferreira et al., 2015). Moreover, it is important to note substantial individual variability in cognitive aging (Hedden and Gabrieli, 2004). It has been argued that increased variability with age is an indication of pathological processes (Hedden and Gabrieli, 2004). Three types of variability are of special interest: *across-individuals variability* (often referred to as individual differences); *across-tasks variability* (the dedifferentiation hypothesis, which postulates that covariance among cognitive variables increases with age); and *across-time variability* (variability within individuals in longitudinal designs) (Hedden and Gabrieli, 2004). To our knowledge, only Zimprich and Mascherek (2010) have studied variability in a middle-age cohort. These authors found that although age-related decline occurred in processing speed, memory, and fluid intelligence, only variance in processing speed increased across time.

The determinants of cognitive variability may be multiple and little research has addressed this issue specifically in middle-age adults. A factor that could contribute to this variability is presence of individuals in the preclinical stage of AD (or any other neurodegenerative disease) in studies of normal aging (Nettiksimmons et al., 2010; Aguilar et al., 2014). Preclinical AD individuals may have subtle cognitive impairment and have increased risk of future cognitive decline supported by amyloid pathology and neurodegeneration (Sperling et al., 2011). Neurodegeneration can be studied through several imaging markers. Global brain atrophy and measurements of cortical thickness and hippocampal volume are frequently used in studies of normal aging and neurodegenerative diseases such as AD (Frisoni and Delacourte, 2009; Sperling et al., 2011). Mean diffusivity (MD) has recently received special attention because it seems to precede gray matter changes in cognitively normal individuals with familial AD (Li et al., 2015) and sporadic AD (Li et al., 2013). White matter hyperintensities, a surrogate marker of cerebrovascular disease, have been related to lower cognitive performance during middle-age (Nunley et al., 2015; Hawkins et al., 2017). Another possible determinant of cognitive variability is inclusion of individuals with subjective cognitive complaints (SCD), which has also been found to confer risk for cognitive decline (Abdulrab and Heun, 2008; Reisberg and Gauthier, 2008). Among different cognitive complaints, memory complaints have been related to increased likelihood of preclinical AD in individuals with SCD (Jessen et al., 2014; Ferreira et al., 2017a). The relationship between subjective memory complaints and objective memory impairment, however, varies from study to study, with both positive and negative results (Crumley et al., 2014). Other factors such as gender, cognitive reserve, and subclinical depressive symptomatology might also contribute to cognitive variability during middle-age. Regarding gender, although higher prevalence of dementia among females is often reported, this is true for diseases such as AD, whereas prevalence of diseases such as vascular dementia is higher in men (Podcasy and Epperson, 2016).

To the best of our knowledge, the role of these factors in cognition during healthy middle-age has not previously been investigated in the same cohort and study. The main aim of this study was to investigate potential determinants of age-related cognitive variability in middle-age adults. We hypothesized that not all middle-age individuals age in the same way *(across-individuals variability)*. In particular, we hypothesized that deviation in cognitive performance from a reference younger group will be related to greater global brain atrophy, smaller hippocampal volume, reduced cortical thickness in association areas, increased MD in white matter tracks connecting these association areas, higher burden of white matter hyperintensities, higher depressive symptomatology, presence of memory complaints, and female gender. Cognitive reserve will play a role, i.e., individuals with high cognitive reserve will deviate less from a reference younger group and will endure better the impact of these factors on cognitive performance. We also hypothesized that variability in cognition will increase with aging, especially in processing speed, as earlier described by Zimprich and Mascherek (2010), and perhaps in executive functions, as suggested in our previous studies (Ferreira et al., 2014a, 2015) (*across-tasks variability*).

## Materials and Methods

### Participants

The purpose of the current study was to investigate variability in cognition during middle-age. To that end, we recruited healthy middle-age adults belonging to two age groups: 40 ± 2 years (including individuals from 38 to 42 years of age, *n* = 30, 12 females and 18 males) and 50 ± 2 years (from 48 to 52 years of age, *n* = 31, 19 females and 12 males). The youngest 40 ± 2 group served as reference and the oldest 50 ± 2 group as target for investigating age-related variability in cognition. Inclusion criteria were: (1) Preserved global cognition and functional status operationalized as a Mini-Mental State Examination (MMSE) score ≥ 26, a Blessed Dementia Rating Scale (BDRS) score < 4, and a Functional Activities Questionnaire (FAQ) score < 6; (2) Normal cognitive performance in comprehensive neuropsychological assessment using pertinent clinical normative data and excluding individuals with performance below 2 SD using own sample descriptive values (i.e., individuals did not fulfill cognitive criteria for mild cognitive impairment), as previously done (Ferreira et al., 2016); (3) No abnormal findings such as stroke, tumors, hippocampal sclerosis, etc., in magnetic resonance imaging (MRI) according to an experienced neuroradiologist; and (4) No psychiatric or neurologic disorders, systemic diseases with neuropsychological consequences, or history of substance abuse, screened out on an exhaustive interview and supported by a Geriatric Depression Scale (GDS, 15-items version) score ≤ 6 and/or a Beck Depression Inventory (BDI, 21-items version) score ≤ 12. This study was carried out in accordance with the recommendations of the local ethics committee of the University of La Laguna (Spain). All participants gave written informed consent in accordance with the Declaration of Helsinki. The protocol was approved by the local ethics committee of the University of La Laguna (Spain).

### Cognitive Assessment

A comprehensive neuropsychological protocol was applied covering the following cognitive domains: cognitive processing speed, motor processing speed, attention, executive functions, premotor functions, episodic memory, visuoconstructive, visuoperceptive and visuospatial functions (visual abilities), and language (**Table 1**). Protocol and administration procedures are fully described in Supplementary Table 1.

**Table 1:** Cognitive domains and variables included.

**COGNITIVE PROCESSING SPEED**	**MEMORY**
**SIMPLE TASKS**	**Immediate recall:**
CTT-1	Logical Memory immediate ^(Verbal)^
PC-Vienna cognitive reaction time	TAVEC learning total ^(Verbal)^
	8/30 SRT learning total ^(Visual)^
	Visual Reproduction immediate ^(Visual)^
**COGNITIVE PROCESSING SPEED**	**Delayed recall:**
**COMPLEX TASKS**	
Stroop sheet 1	Logical Memory delayed ^(Verbal)^
Stroop sheet 2	TAVEC delayed ^(Verbal)^
TDAS-Nouns time	8/30 SRT delayed ^(Visual)^
TDAS-Actions time	Visual Reproduction delayed ^(Visual)^
TGAAS time	
**MOTOR PRECESSING SPEED**	**Recognition:**
PC-Vienna motor reaction time	Logical Memory recognition ^(Verbal)^
Block Design time control task (simple)	TAVEC recognition ^(Verbal)^
Block Design time control task (complex)	8/30 SRT recognition ^(Visual)^
	Visual Reproduction recognition ^(Visual)^
**ATTENTION**	**VISUOCONSTRUCTIVE, VISUOPERCEPTIVE AND VISUOSPATIAL FUNCTIONS (VISUAL ABILITIES)**
PASAT	FRT
Digit Span forward score	Visual Reproduction visual discrimination
Visuospatial Span forward score	Blocks Design total WAIS-III score
	Visual Reproduction copy
	JLOT 1
	JLOT 2
**EXECUTIVE FUNCTIONS**	**LANGUAGE**
Stroop interference index	TDAS-Nouns correct responses
Verbal fluency (letters)	TDAS-Actions correct responses
Verbal fluency (animals)	TGAAS correct responses
Verbal fluency (actions)	
Digit Span backward score	
Visuospatial Span backward score	
CTT (1 minus 2)	
**PREMOTOR FUNCTIONS**	
Luria’s hand alternative movements	
Luria’s motor coordination	
Luria’s motor inhibition	

*CTT, Color Trails Test; TDAS, Nouns and Actions Naming Test (TDAS: *Test de Denominación de Acciones y Sustantivos*); TGAAS, Generating Actions by Semantic Association (TGAAS: *Test de Generación de Acciones por Asociación Semántica*); PASAT, Paced Auditory Serial Addition Test; TAVEC, Spanish version of the California Verbal Learning Test (CVLT) (TAVEC: *Test de Aprendizaje Verbal España-Complutense*); SRT, Spatial Recall Test; FRT, Facial Recognition Test; WAIS-III, Wechsler Adult Intelligence Scale – Third Edition; JLOT, Judgment of Line Orientation Test.*

### Subjective Memory Complaints, Cognitive Reserve, Depressive Symptomatology, and Gender

Subjective memory complaints were elicited by the question “*Do you have difficulties with your memory?*” This question referred to changes observed during the last 6 months. Absence of complaints was coded as 0 and presence of complaints was coded as 1. The WAIS-III Information subtest (Wechsler, 1997) was used as a proxy of cognitive reserve. WAIS-III Information better represents achievements and/or usage of educative opportunities in comparison with educational attainment or years of education in populations that received heterogeneous formal education (Correia et al., 2015), as the one included in this study. Moreover, among several reserve proxies, WAIS-III Information showed the greatest compensation capacity of the effect of cortical thinning on cognition (Ferreira et al., 2016). Scores in WAIS-III Information range from 0 to 28, with higher values reflecting greater capacity. Depressive symptomatology was measured with the 15-items version of the GDS and/or the 21-items version of the BDI. The original scores from both scales were Z-transformed and combined into a single measure. Gender was recorded and coded as 0 female and 1 male.

### Magnetic Resonance Imaging (MRI)

Participants were scanned using a 3.0T General Electric imaging system (General Electric, Milwaukee, WI, United States) located at the *Hospital Universitario de Canarias* in Tenerife, Spain. A three-dimensional T1-weighted FSPGR (Fast Spoiled Gradient Echo) sequence, a three-dimensional FLAIR (Fluid Attenuation Inversion Recovery) sequence, and a DTI (Diffusion Tensor Imaging) sequence were acquired in sagittal, sagittal and axial plane, respectively. The parameters applied were as follows, T1-weighted: repetition time/echo time/inversion time = 8.73/1.74/650 ms, field of view 250 mm × 250 mm, matrix 250 mm × 250 mm, flip angle 12°, slice thickness = 1 mm; FLAIR: repetition time/echo time = 6500/≈450 ms, field of view 250 mm × 250 mm, matrix 256 mm × 256 mm, slice thickness = 1.5 mm; DTI: repetition time/echo time = 15000/≈72 ms., field of view 256 mm × 256 mm, matrix 128 mm × 128 mm, 31 directions, B value = 1000, flip angle 90°, slice thickness = 2.4 mm. Full brain and skull coverage was required for the MRI datasets and detailed quality control was carried out on all MR images according to previously published criteria (Simmons et al., 2009).

The T1-weighted images were processed and analyzed with the FreeSurfer 5.1.0 image analysis suite^1^ (see Supplementary Table 2 for full details and references). This procedure has previously been used for imaging-neuropsychological analysis (Liu et al., 2011), imaging-cerebrospinal fluid analysis (Ferreira et al., 2014b), biomarker discovery (Thambisetty et al., 2011), and generation of a multivariate diagnostic index for AD diagnosis and SCD and MCI prediction (Westman et al., 2010; Aguilar et al., 2014; Ferreira et al., 2017a). The measures of hippocampal volume (left + right) and total volume of the ventricles (estimation of global brain atrophy) were corrected for intracranial volume and selected for the analysis. Cortical thickness across the cortical mantle was also analyzed at the vertex level. White matter hyperintensities were segmented and lesion load calculated with CASCADE^2^, an in-house technique described elsewhere (Damangir et al., 2012, 2016). Briefly, the procedure uses T1 and FLAIR images. First, pre-processing co-registers T1 and the brain tissue segmentation from FreeSurfer to FLAIR space before applying inhomogeneity correction and noise removal. Then white matter hyperintensities are measured using a non-parametric statistical test that captures all area whose local intensity is significantly brighter than normal appearing white matter on the FLAIR image. Post-processing removes small detection and detections in unlikely areas before measuring the total lesion load. DTI data were processed and analyzed with the FSL software^3^, using the FDT and TBSS tools (see Supplementary Table 3 for full details and references). The measure of MD was selected for the analysis. Careful visual quality control was performed on all the data obtained from both FreeSurfer and FSL, and manual edits were done when appropriate.

### Statistical Analysis

**FIGURE 1:**
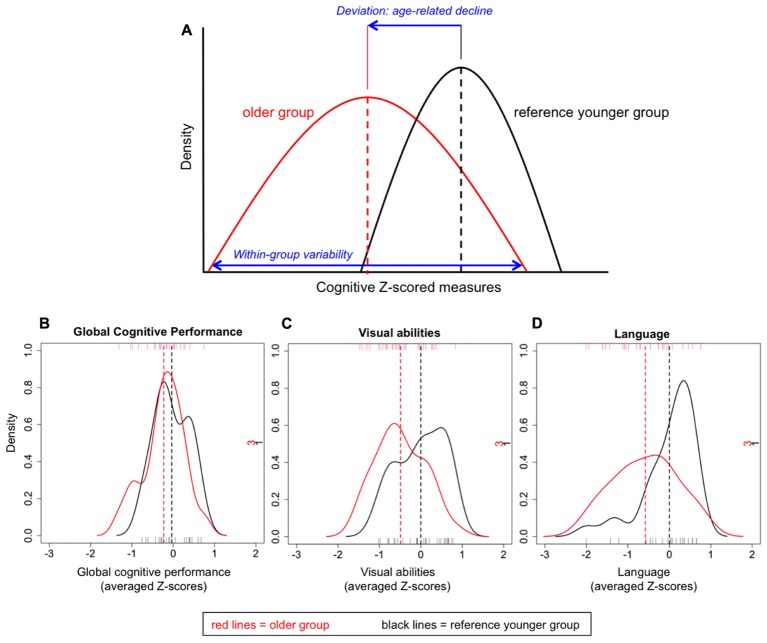
Graphical example of the cognitive *Z*-scored measures **(A)** and density plots for the indexes of global cognitive performance **(B)**, visual abilities **(C)**, and language **(D)**.

Variability in cognition during middle-age was studied by comparing performance of the older group (50 ± 2) versus the reference younger group (40 ± 2). First, we selected the reference younger group and transformed all their cognitive measures to *Z*-scores using means and standard deviations from the reference younger group itself. These *Z*-scored measures were then combined and averaged as detailed in **Table 1** in order to create nine cognitive domains, also reducing the number of statistical tests. An index of global cognitive performance was also calculated by averaging these nine cognitive domains. We thus obtained *Z*-scores for all the cognitive measures, nine cognitive domains, and an index of global cognitive performance for the reference younger group. Second, we selected the older group and transformed all their cognitive measures to *Z*-scores using again means and standard deviations from the reference younger group. These *Z*-scored measures were also combined to create nine cognitive domains and an index of global cognitive performance. For the older group, all these *Z*-scored measures thus refer to deviation of cognitive performance with reference to the younger group, in other words, to the trend of cognitive decline (within normality) after a 10-year period. Furthermore, variability within the older group is also retained in all these measures (see **Figure 1A** for a graphical example). For this reason, the index is labeled ‘global deviation index’ when it refers to the older group. Hence, in the results section, our first hypothesis (*across-individuals variability*) is addressed when reporting correlations, and our second hypothesis (*across-tasks variability*) is addressed when reporting average deviation. Individuals with missing values in some of the cognitive variables were excluded only for analyses involving the index of global cognitive performance (sample size: 50 ± 2 group, *n* = 23; reference group, *n* = 22). Most of the analyses reported in this manuscript are based on the older group, otherwise specified.

MANOVA and independent one-way ANOVA or *T*-test were used for between-group mean comparisons and dependent one-way ANOVA for within-group mean comparisons. The Chi-square test was used for categorical variables. Pearson correlations and partial correlations were used to study relationships between variables. Effect sizes are reported as partial eta squared ($\eta_{p}^{2}$) or correlation coefficients (*r*).

The Benjamini–Hochberg’s correction (Hochberg and Benjamini, 1990) for multiple testing was applied in all statistical analyses, using a *p*-value < 0.05 (two-tailed) as significant. These statistical analyses were performed using SPSS 22.0 for Mac.

Vertex analyses across the cortical mantle were conducted using the FreeSurfer software. Maps were smoothed using a circularly symmetric Gaussian kernel across the surface with a full width at half maximum (FWHM) of 15 mm. A general linear model was fitted at each vertex using cortical thickness as dependent variable. Results were tested against an empirical null distribution of maximum cluster size across 5.000 iterations. Z Monte Carlo simulations were used with a cluster-forming threshold of *p* ≤ 0.05 (two-sided for primary analyses, one-sided for follow-up analyses), yielding clusters corrected for multiple comparisons across the cortical mantle. Voxel-based analyses on the white matter skeleton were performed using the FSL software. A general linear model was fitted at each voxel using MD as dependent variable. Permutation-based non-parametric testing with 5000 iterations was used followed by threshold-free cluster enhancement (TFCE) and family-wise error (FWE) multiple comparisons correction (*p* ≤ 0.05, two-sided). Exploratory analyses were also performed with uncorrected *p* ≤ 0.001 deemed significant.

## Results

The older group (50 ± 2) was statistically comparable to the reference younger group (40 ± 2) in gender distribution, WAIS-III Information subtest, and all clinical tests (**Table 2**).

**Table 2:** Demographic and clinical variables.

	Age 40 ± 2 (*n* = 30)	Age 50 ± 2 (*n* = 31)	*p*-value
Age, years	40.33 (1.30)	49.84 (1.29)	**<0.001**
Gender, % female	40	61	0.576
WAIS-III Information	15.03 (5.37)	15.97 (5.54)	0.883
MMSE	29.33 (1.06)	28.90 (1.04)	0.580
BDRS ^a^	0.48 (0.89)	0.45 (0.86)	0.883
FAQ	0.33 (0.66)	0.58 (0.85)	0.654
BDI ^b^	3.62 (2.90)	5.64 (3.42)	0.576
GDS ^b^	1.47 (1.46)	2.22 (1.39)	0.654


*Values in the table represent mean (SD) except for gender where percentage is displayed. All *p*-values are adjusted using the Benjamini–Hochberg’s correction for multiple testing. Significant results are highlighted in bold for easier reading. ^a^*n* = 60; ^b^Depressive symptomatology was measured with BDI (*n* = 35) or GDS (*n* = 26). WAIS-III, Wechsler Adult Intelligence Scale – Third Edition; MMSEI, Mini-Mental State Examination; BDRSI, Blessed Dementia Rating Scale; FAQI, Functional Activities Questionnaire; BDII, Beck Depression Inventory; GDSI, Geriatric Depression Scale.*

### Across-Individuals Variability in Cognition

**FIGURE 2:**
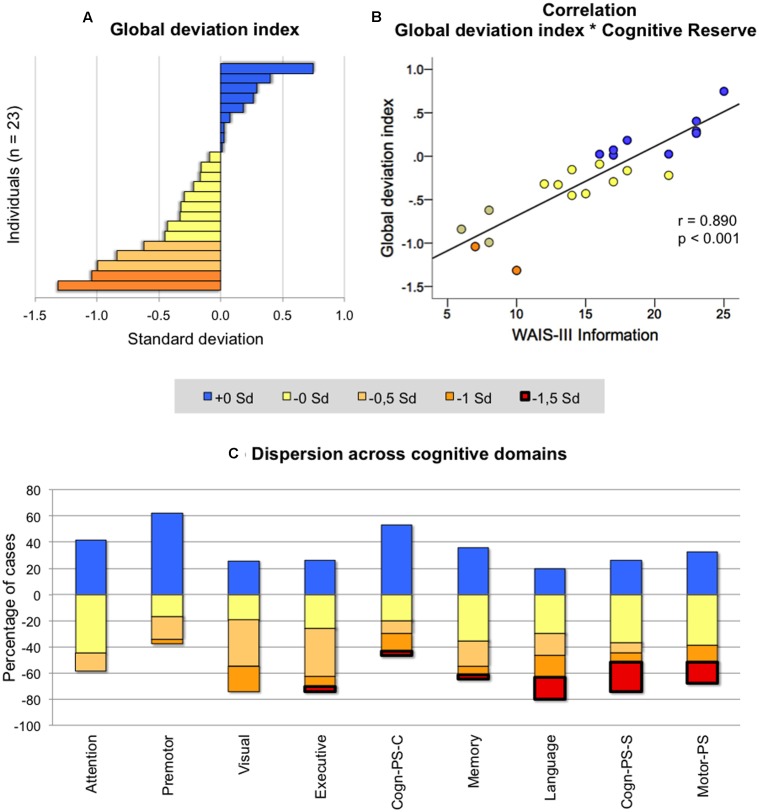
Across-individuals variability in cognition. Deviation in individuals of age 50 ± 2 from the reference group (age 40 ± 2) was categorized in five levels depending on the degree of deviation (+0 SD = positive deviations indicating better performance in the group of age 50 ± 2; -0 SD = negative deviations from -0.01 to -0.50 SD; -0.5 SD = negative deviation from -0.51 to -1.00 SD; -1 SD = negative deviation from -1.01 to -1.50 SD; -1.5 SD = negative deviation larger than -1.51. All negative deviations indicate worse performance in the group of age 50 ± 2). The *y*-axis in **(C)** represents the percentage of cases showing positive and negative deviation across cognitive domains. For instance, in attention, 40 means that 40% of the individuals showed a positive deviation, while -60 means that 60% of the individuals showed a negative deviation from the reference group. Analyses in **(A,B)** were performed on 23 individuals, and analyses on **(C)** included the whole group of age 50 ± 2 (*n* = 31). WAIS-III, Wechsler Adult Intelligence Scale – Third Edition; PS, processing speed; Cogn-PS-S, cognitive processing speed simple tasks; PS-C, cognitive processing speed complex tasks; Visual, visuoconstructive, visuoperceptive and visuospatial functions.

Across-individuals variability in cognition during middle-age was analyzed in order to investigate whether all 50 ± 2 years old individuals deviated in the same extent from the reference younger group. For this analysis, *Z*-scores from both age groups were compared. The index of global cognitive performance was statistically comparable between the two groups [*t*_(43)_ = -1.410; *p* = 0.166; **Figure 1B**], indicating that on average, older individuals do not statistically deviate from the reference group. When focusing on the older group, certain variability can be observed at the individual level (**Figure 2A**). Although most individuals (78%) had a global deviation index around 0 SD, two individuals exceeded -1 SD. Nine participants (39%) evidenced a positive global deviation index.

We then looked at the nine cognitive domains in order to investigate whether age-related differences were heterogeneous across cognitive functions in the older group (**Figure 2C**). Attention was the function where individuals showed less negative dispersion (i.e., less red-yellow colors in **Figure 2C**; range from 0 to -1 SD), followed by premotor and visual abilities (range from 0 to -1.5 SD). In all other cognitive domains, dispersion of negative deviations was greater and exceeding -1.5 SD.

The correlation between the global deviation index and WAIS-III Information, gender, subjective memory complaints, and the different neuroimaging markers was tested in order to investigate possible determinants of cognitive variability in the older group. Associations with BDRS, FAQ, and depressive symptomatology were also investigated to further characterize variability in global cognition despite clinical depression and functional impairment (BDRS and FAQ) were excluded from the study design. Greater negative deviation from the reference group (lower global deviation index) was strongly correlated to lower values on WAIS-III Information (**Figure 2B**), and showed a trend for a correlation with smaller hippocampal volume and female gender (**Table 3a**). However, no statistical evidence was found for the correlation between the global deviation index and subjective memory complaints, depressive symptomatology, global brain atrophy or white matter hyperintensities. Interestingly, WAIS-III Information influenced the correlations with gender and hippocampal volume by removing the significant effect when this was included as a covariate (**Table 3b**). The same correlations were also tested for visual abilities and language, which were the only cognitive domains where the older group significantly deviated from the reference younger group (see results further down in section “Across-Tasks and Across-Domains Variability in Cognition”). Regarding visual abilities, the pattern of significant uncorrected results almost perfectly replicated the results for the global deviation index described above (**Table 3a**). Again, WAIS-III Information exerted a strong modulatory effect by removing all the significant effects obtained at the uncorrected level (**Table 3b**). Regarding language, greater negative deviation from the reference group (lower performance) was correlated with higher values in both BDRS and FAQ (**Table 3a**). However, language results did not survive the Benjamini–Hochberg’s correction for multiple testing.

**Table 3:** Association between cognitive measures and the demographic, clinical and neuroimaging variables.

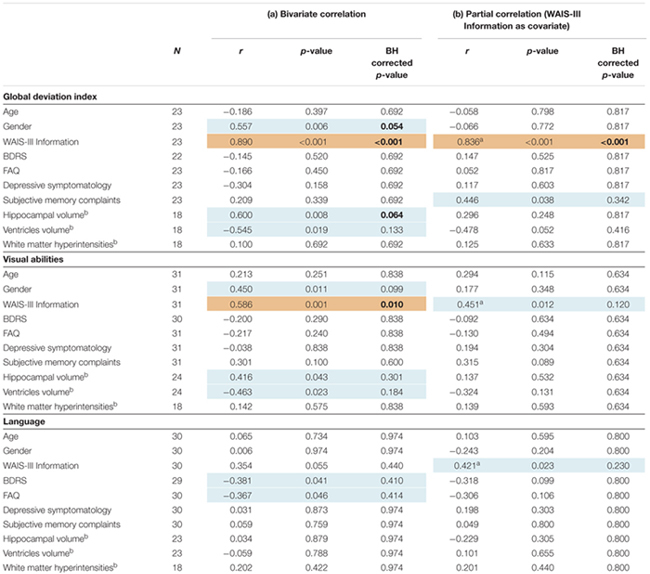

*These correlations were performed in the older group. Values for the global deviation index were available for 23 individuals. Values for visual abilities were available for the whole older group (*n* = 31). Values for language were available for 30 individuals. In addition, there were missing data for BDRS (one missing case), and the imaging measures (five missing cases). Due to variability in sample size, we replicated the correlations for visual abilities and language in the same sample than for the analyses on the global deviation index (*n* = 23). The pattern of results was the same (data not shown). Since depressive symptomatology was measured with BDI (*n* = 22) or GDS (*n* = 9), original scores were Z-transformed in order to combine both measures. Gender was coded as 0 female and 1 male. Significant BH corrected results (or trends) are highlighted in bold for easier reading. Patterns of significant uncorrected results are shadowed in light blue (or light brown when results remain significant after the BH correction). ^a^Partial correlation, covariate: gender; ^b^All volumetric imaging variables are corrected for intracranial volume. BH corrected *p*-value = *p*-values are adjusted using the Benjamini–Hochberg’s correction for multiple testing; WAIS-III, Wechsler Adult Intelligence Scale - Third Edition; BDRS, Blessed Dementia Rating Scale; FAQ, Functional Activities Questionnaire.*

**FIGURE 3:**
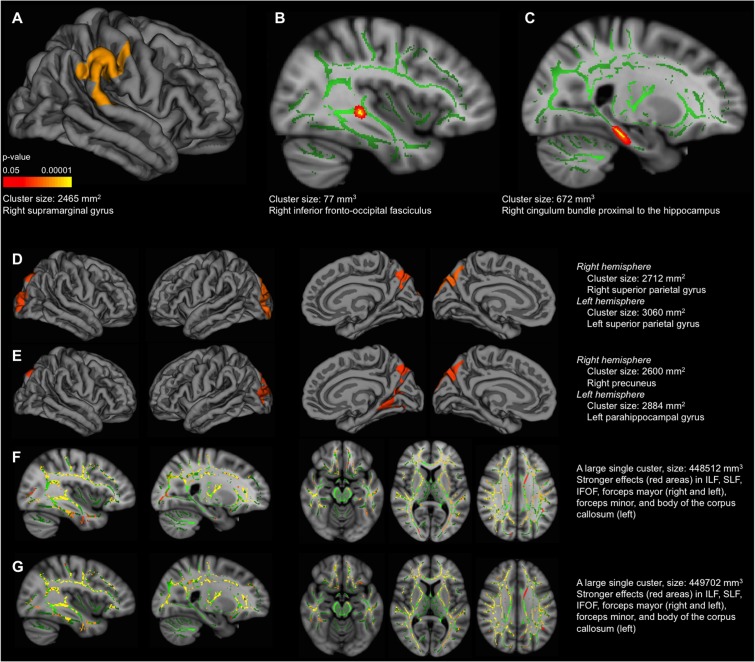
Correlation between cognitive measures and cortical thickness/mean diffusivity (MD). These correlations were performed in the older group. MRI data and values for the global deviation index were available for 18 individuals. MRI data and values for visual abilities were available for 24 individuals. MRI data and values for language were available for 23 individuals. The results displayed in the figure correspond to the sample based on the global deviation index (*n* = 18) to allow comparability across the three cognitive measures. The same correlations were replicated for visual abilities and language in 24 and 23 individuals, respectively. The pattern of results was similar for MD, although significant correlations were mainly found in the right hemisphere; and results did not survive the Z Monte Carlo cluster-forming threshold for cortical thickness, although similar results were observed at the uncorrected level (data not shown). Cortical thickness **(A,D,E)**: lateral view of the right hemisphere in **(A)**. Lateral view of the right and left hemispheres (first and second images from left, respectively), and medial view of the right and left hemispheres (third and fourth images from left, respectively), in both **(D,E)**. Correlation with the global deviation index in **(A)**, correlation with visual abilities in **(D,E)**, including WAIS-III Information as covariate in **(E)**. Significant vertexes are displayed as colored scale (cluster-forming threshold yielding clusters corrected for multiple comparisons) with red–yellow indicating a positive correlation in **(A**,**D,E)**. MD **(B,C,F,G)**: **(B)** intermediate view (*x* coordinate = 51 mm) of the right hemisphere. Significant voxels are displayed in red–yellow (FWE corrected), showing a negative correlation. Significant voxels were thickened for easier visualization; **(C)** intermediate view (*x* coordinate = 66 mm) of the right hemisphere. Significant voxels are displayed in red (uncorrected *p* < 0.001), showing a negative correlation. Significant voxels were thickened for easier visualization; **(F,G)** intermediate view (first and second images from left, *x* coordinates = 51 and 66 mm, respectively) of the right hemisphere in sagittal plane; intermediate view (third, fourth, and fifth images from left, *z* coordinates = 56, 79 and 102 mm, respectively) in axial plane. In all **(F,G)** images, significant voxels are displayed in red–yellow (FWE corrected), showing a negative correlation. Correlation with the global deviation index in **(B,C)**, correlation with visual abilities in **(F,G)**, including WAIS-III Information as covariate in **(G)**. The green regions in **(B)**, **(C)**, **(F)** and **(G)** represent the mean white matter skeleton. ILF, inferior longitudinal fasciculus; SLF, superior longitudinal fasciculus; IFOF, inferior frontal–occipital fasciculus. All the regions labeled in the figure correspond to the cluster maximas (e.g., right supramarginal gyrus).

The vertex-based analysis showed that greater negative deviation from the reference group (lower global deviation index) was associated with less cortical thickness in the right parietal–temporal–occipital association cortex, extending to a small area on the postcentral gyrus (analysis conducted in the older group) (**Figure 3A**). This association was no longer significant once accounting for WAIS-III Information. Regarding white matter integrity, the voxel-based analysis showed that greater negative deviation from the reference group was associated with increased MD in the right inferior fronto-occipital fasciculus (**Figure 3B**, FWE corrected) and cingulum bundle proximal to the hippocampus (analysis conducted in the older group) (**Figure 3C**, uncorrected). Again, this association was no longer significant once accounting for WAIS-III Information. The same imaging correlations were also tested for visual abilities and language. Regarding visual abilities, greater negative deviation from the reference group (lower performance) was associated with less cortical thickness in lateral and medial parietal–occipital regions (**Figure 3D**), and increased MD in association white matter tracks connecting these with the frontal and temporal lobes (**Figure 3F**). These associations remained significant after accounting for WAIS-III Information (**Figures 3E,G**). No significant correlations were obtained for language.

### Across-Tasks and Across-Domains Variability in Cognition

All the following analyses are conducted in the older group, otherwise specified. We wanted to study whether cognitive performance in the older group deviated from the reference younger group in the same extent across cognitive tasks or domains. A dependent one-way ANOVA was performed including the nine cognitive domains as within-subject factor. Results showed significant across-domains variability [*F*_(4,82)_ = 4.269; *p* = 0.004; $\eta_{p}^{2}$ = 0.163]. A descriptive analysis showed that all domains except premotor functions (+0.10 SD) deviated negatively from the reference group, never exceeding -1 SD. Language evidenced the greatest deviation (-0.58 SD), followed by cognitive processing speed in simple tasks (-0.54 SD), visual abilities (-0.49 SD), motor processing speed (-0.41 SD), executive functions (-0.34 SD), memory (-0.24 SD), attention (-0.02 SD), and cognitive processing speed in complex tasks (-0.01 SD). A MANOVA was performed including the age group as between-subject factor (50 ± 2 vs. 40 ± 2) and the nine cognitive domains as dependent variables. Results showed that the older group deviated significantly from the reference younger group [*F*_(9,35)_ = 3.115; *p* = 0.007; $\eta_{p}^{2}$ = 0.445]. Follow-up analyses showed that only deviation in language (*p* = 0.024; *r* = -0.383) and visual abilities (*p* = 0.018; *r* = -0.394) was significant (**Figures 1C,D**, respectively). Since memory is a complex cognitive function, and previous studies have shown that aging exerts a stronger effect on free recall than on recognition (Luo and Craik, 2008), we further subdivided the memory domain into immediate recall, delayed recall, and recognition. A descriptive analysis showed that immediate recall showed the greatest deviation from the reference group (-0.33 SD), followed by delayed recall (-0.22 SD), and recognition (-0.18 SD).

**FIGURE 4:**
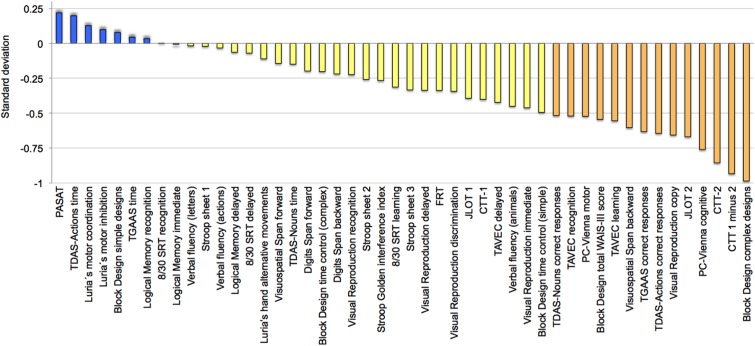
Across-tasks variability in cognition. Cognitive performance in the group of age 50 ± 2 (*n* = 31) ordered according to deviation from the reference group (age 40 ± 2). Negative deviation means worse performance in the older group. See full name of all cognitive measures in **Table 1** and Supplementary Table 1.

To complement these findings, a follow-up descriptive analysis was performed in order to explore variability across cognitive tasks in the older group. All cognitive variables were ordered based on the magnitude of the deviation (**Figure 4**). Eight variables (17%) deviated positively, although never exceeding +0.25 SD. All the other variables deviated negatively, with block design, CTT, PC-Vienna, JLOT 2, visual reproduction copy, TDAS-Actions, TGAAS correct responses, visuospatial span backward, and TAVEC learning showing greater deviation.

## Discussion

The findings of this study show that age-related cognitive differences do not occur in the same magnitude and direction in all middle-age individuals. Deviation in global cognition from a reference younger group was not statistically significant. This is in line with what is expected in normal aging, but there was variability with some individuals deviating more than -1 SD. When breaking global cognition down to different cognitive domains, the distribution of negative deviations was rather homogeneous in attention, premotor functions, and visual abilities. For all other cognitive domains the deviation was greater, especially in cognitive processing speed in simple tasks, language, and motor processing speed, with individuals exceeding -1.5 SD. These results may be of clinical interest for interpretation of deviation from normality in certain neuropsychological tests, which is not well documented in the literature yet, especially in middle-age. To our knowledge, only Zimprich and Mascherek (2010) have provided data on across-individuals variability in middle-age subjects. These authors reported that across-individuals differences increased over time in processing speed, in line with our results. In contrast, differences remained unchanged in memory. Our results show that when different components are studied, deviation in memory is not homogeneous, with immediate recall showing greater deviation, followed by delayed recall and recognition.

Regarding possible determinants of variability, greater deviation in global cognition was associated with regional brain atrophy (reduced cortical thickness in the right parietal–temporal–occipital association cortex, and increased MD in the right inferior fronto-occipital fasciculus). Exploratory uncorrected results also showed an association with increased MD in the cingulum bundle proximal to the hippocampus. A trend was also observed for smaller hippocampal volume. The parietal–temporal–occipital association cortex, cingulum bundle, and hippocampus are part of the so-called default mode network (DMN) (Buckner et al., 2008). The medial temporal lobe subsystem of the DMN supports memory and the medial prefrontal subsystem facilitates flexibility. Both subsystems converge in the posterior cingulate cortex, important for integration, and are connected through white matter tracts such as the cingulum bundle (Buckner et al., 2008). Aberrant DMN is well documented in normal aging as well as neurodegenerative disorders such as AD, and less deactivation has been found to be modulated by higher levels of cognitive reserve (Bartrés-Faz and Arenaza-Urquijo, 2011). The right inferior fronto-occipital fasciculus connects occipito-parietal regions with the ventrolateral prefrontal cortex, a region involved in memory encoding (Wagner et al., 1998). White matter integrity in cingulum and the inferior fronto-occipital fasciculus becomes abnormal prior to cognitive decline in individuals at high risk of AD (Gold et al., 2012). Also, aging has been associated with less connectivity in fronto-parietal and fronto-occipital systems in healthy old adults (Sala-Llonch et al., 2014). Therefore, the association between variability in global cognition and deterioration in these brain regions might be interpreted as an early sign of degeneration perhaps leading to development of future pathological conditions such as AD. This remains, however, at the speculative level and future studies including functional MRI resting state and longitudinal designs may add relevant information, particularly in regard to the networks discussed above.

Complementary analysis on visual abilities and language, which were the only cognitive domains that significantly deviated from the reference younger group, showed that lower performance in visual abilities was associated with less cortical thickness in parietal–occipital regions, and increased MD in association white matter tracks connecting these with the frontal and temporal lobes. The occipital and parietal cortex is involved in visual and spatial processing, and the tasks included in the visual abilities index have been related to frontal–parietal, frontal–occipital, and temporal–occipital networks (Ng et al., 2000; Tranel et al., 2009; Biesbroek et al., 2014; Frässle et al., 2016). These associations remained significant after accounting for WAIS-III Information, which is coherent with the finding that cognitive reserve is seldom related to the occipital cortex (Bartrés-Faz and Arenaza-Urquijo, 2011; Steffener and Stern, 2012). No significant correlations were obtained for language, perhaps because difficulties in lexical access (the main function included in the language index) have been reported to remain stable during middle-age (Tombaugh and Hubley, 1997; Kent and Luszcz, 2002).

Greater deviation in global cognition and visual abilities was strongly associated with lower cognitive reserve, as measured by the WAIS-III information subtest, and the associations described above were mostly explained by variability in cognitive reserve. Previous studies have demonstrated that cognitive reserve is a strong modulator of disease progression, delaying the appearance of clinical symptoms secondary to brain pathology (Stern, 2009; Sperling et al., 2011). Our results suggest a protective role of cognitive reserve during middle-age, i.e., individuals with high cognitive reserve performed better in cognitive tests and deviated less from a reference younger group. In addition, individuals with high cognitive reserve also endured better the effect of cortical thinning and increased MD on global cognition. Thus, not only protective but also compensatory effects could be taking place already before the age of 50. Indeed, a recent study showed that cognitive reserve buffers the effect of cortical thinning on cognition during middle-age (Ferreira et al., 2016). Greater deviation in global cognition and visual abilities also showed a trend for the correlation with female gender. The prevalence of dementia is often higher in females, but in diseases such as vascular dementia the reported pattern is opposite (Podcasy and Epperson, 2016). However, in the current study, greater deviation in global cognition in females was explained by lower cognitive reserve in females, and therefore possibly not by gender *per se*.

No significant associations were obtained between deviation in global cognition and subjective memory complaints, depressive symptomatology, or white matter hyperintensities. Other studies have reported no associations between subjective memory complaints and performance on objective memory tests, especially in younger old adults (Crumley et al., 2014). Although depressive symptomatology is known to influence cognition, the lack of correlation in our study may be explained by exclusion of individuals with clinical depression. Instead, this variable only reflected variability in depressive symptomatology within the normal range, that is, subclinical depressive symptomatology (none of the participants in this study were taking antidepressants). White matter hyperintensities have been related to lower cognitive performance during middle-age in samples with high vascular risk (Nunley et al., 2015; Hawkins et al., 2017). However, this association has not been obtained in samples excluding vascular pathology (Postma et al., 2016), consistent with our study.

This study also shows that age-related cognitive differences do not occur in the same magnitude and direction across cognitive functions and tasks. Literature on cognition during middle-age is not conclusive at present and evidence usually comes from samples including broad age ranges rather than specific middle-age studies (Hedden and Gabrieli, 2004; Willis et al., 2010). Recently, age-related differences in cognition were found to be extensive in the transition from middle-age to old age, and only subtle cognitive dysfunction was observed before the age of 50 (Ferreira et al., 2015). In particular, early middle-age (40 to 50 years of age) was characterized by decline in cognitive processing speed, executive control, initial learning in verbal episodic memory, complex visuoconstructive and visuospatial functions, and lexical access by semantic associations (Ferreira et al., 2015). Of note, this early cognitive decline was mediated by age-related differences in several gray matter regions (Ferreira et al., 2014a). Other groups have also reported early executive dysfunction and slowing in processing speed during middle-age (Salthouse, 2009; Zimprich and Mascherek, 2010; Singh-Manoux et al., 2012).

In the current study, our descriptive analysis revealed that, in addition to the functions described above, motor reaction time, learning on episodic memory (both verbal and visual), and lexical access by visual confrontation were among the most deviated cognitive tasks. Taken these two studies together, our findings suggest that regarding processing speed, the cognitive modality may be affected earlier and motor aspects may be affected later. To our knowledge, this temporality has not been studied before and deserves replication in a larger sample. This may help to define the very early stage of well-documented processing slowing in the old age (Baltes et al., 1999; Finkel et al., 2003; Meijer et al., 2009; Schaie, 2009; Zimprich and Mascherek, 2010). Our results in episodic memory support a pattern of early decline in learning, with later appearance of difficulties in free retrieval in the transition from middle-age to the old age (Ferreira et al., 2015), and normal performance in consolidation until the very old age (Luo and Craik, 2008). Finally, lexical access by semantic associations seems to occur before lexical access by visual confrontation, possibly due to increased difficulty and involvement of executive functions in the former one. To our knowledge, this is also the first study suggesting such temporality in middle-age. Age-related differences in lexical access has previously been described using different age spans and neuropsychological tests (Barresi et al., 2000; Mackay et al., 2002; Tsang and Lee, 2003). Some of the results discussed in this paragraph are based on the across-tasks variability analysis. The large amount of cognitive variables investigated here is unique in a study of this kind and thus provides valuable information. However, these many variables might raise issues related to multiple testing. For this reason, data on across-tasks variability is presented in a descriptive manner and future studies are needed to further investigate the temporality suggested here. Also, multivariate analyses could be conducted in larger samples in order to investigate the interactive contribution of factors such as gender, depressive symptomatology, cognitive reserve, etc. (Ferreira et al., 2017b). In the current study, we did not find any partial contribution of gender and depressive symptomatology to deviation in the global deviation index. However, whether these factors contribute to deviation in specific cognitive domains and tasks warrants further investigation.

This study is one of the few investigating variability in cognition during middle-age. The inclusion of numerous cognitive measures, different markers of neurodegeneration and cerebrovascular disease, and clinically relevant factors such as subjective memory complaints, cognitive reserve and depressive symptomatology is an advantage of this study. Some limitations should also be considered. The cross-sectional nature of our data allows studying age-related differences, but these results should be complemented with longitudinal studies. Findings in this study should be confirmed in larger cohorts for results generalization. However, large cohorts of early middle-age individuals including comprehensive cognitive data and MRI assessments are scarce in the literature. Neurodegeneration is common in different disorders and studying other pathological markers is needed to fully understand variability in cognitive decline. For example, investigating whether amyloid pathology contributes to cognitive variability in middle-age is warranted to understand implications for early detection of neurodegenerative disorders such as AD. We also studied white matter hyperintensities as a surrogate marker of cerebrovascular disease. Extending our current analysis by exploring how the spatial location of these white matter hyperintensities (and neurodegeneration and amyloid pathology) may contribute to disruption of specific brain networks is of great interest. Investigating that question could contribute to explain variability in specific cognitive domains and/or tasks. Finally, other possible determinants of variability in cognitive aging could not be investigated in this study. Lifestyle and genetic risk factors such as APOE ε4 genotype might be of special interest. In relation to this, systemic diseases such as diabetes, hypothyroidism, and cardiovascular pathology, as well as history of substance abuse, were excluded from this study. These factors increase risk of future cognitive decline and some of them are common in aging, thus warranting further investigation.

## Conclusion

Our results show that variability in cognition during early-middle-age is associated with degeneration of the parietal–temporal–occipital association cortex and white matter tracts connecting this to the prefrontal dorsolateral cortex and hippocampus. This effect is influenced by the cognitive reserve. However, subclinical depressive symptomatology, presence of subjective memory complaints, and white matter hyperintensities do not seem to contribute to variability in cognition during middle-age in this cohort. Although age-related cognitive differences are not extensive during early-middle-age, studying variability offers valuable information showing that differences do not occur in the same magnitude and direction across individuals, cognitive domains and tasks. These findings have at least two important implications. Previous research is based on mean comparisons. However, this could disregard age-related cognitive differences in middle-age since decline is subtle, protective and compensatory mechanisms are still functional, and there is across-individuals variability. This complicates the important task of detecting subtle cognitive impairment, therefore delaying early interventions. A second implication is that as shown, some cognitive measures are much more sensitive to aging, but also, performance in some cognitive measures can be quite heterogeneous across individuals. This is extremely important in the clinical context when it comes to interpretation of deviation from normality in borderline individuals. Although variability seems to be part of middle-age, individuals evidencing borderline performance in cognitive tasks should be further investigated and clinically followed. As shown in the current study, this is especially important in individuals with low cognitive reserve or signs of neurodegeneration in imaging investigations.

## Author Contributions

The study design and concept was done by DF, AN, RC, EW, and JB. Acquisition, analysis, and interpretation of data was done by all the authors. Drafting of the manuscript was done by DF, EW, and JB. Critical revision of the manuscript for important intellectual content was done by all the authors. Statistical analyses were carried out by DF. All the authors provided administrative, technical, and material support. All the authors approved the final version of the article.

## Conflict of Interest Statement

The authors declare that the research was conducted in the absence of any commercial or financial relationships that could be construed as a potential conflict of interest.

## Footnotes

1 http://surfer.nmr.mgh.harvard.edu/

2 http://ki.se/en/nvs/cascade

3 www.fmrib.ox.ac.uk/fsl/
