# Supplementary material for: Cognitive Variability during Middle-Age: Possible Association with Neurodegeneration and Cognitive Reserve
Source: Front Aging Neurosci. 2017 Jun 9;9:188. doi: 10.3389/fnagi.2017.00188 (PMC5465264; doi:10.3389/fnagi.2017.00188)
Supplement: Supplementary file 1 [file Table_1.DOCX]

**Cognitive Variability During Middle-Age: Possible Association With Neurodegeneration And Cognitive Reserve**

Daniel Ferreira*, Alejandra Machado, Yaiza Molina, Soheil Damangir, Antonieta Nieto, Rut Correia, Eric Westman, and José Barroso*

* Correspondence:

- Daniel Ferreira, PhD. Division of Clinical Geriatrics, Center for Alzheimer Research, Department of Neurobiology, Care Sciences and Society, Karolinska Institutet. Novum, Plan 5, 14186, Stockholm (Sweden). Email: [daniel.ferreira.padilla@ki.se](mailto:daniel.ferreira.padilla@ki.se). Telephone: +46720128047. Fax: +46858585470.
- José Barroso, PhD. Faculty of Psychology, University of La Laguna, 38205, Tenerife (Spain). Email: [jbarroso@ull.edu.es](mailto:jbarroso@ull.edu.es). Telephone: +34922317565. Fax: +34922317461.

**Supplemental Data**

**Supplementary Table 1. Neuropsychological protocol: description, administration procedures, and references**

| **Test abbreviation / name in the manuscript** | **Test full name (reference)** | **Variables included in the study – administration** |
| --- | --- | --- |
| *Clinical tests* | | |
| WAIS-III Information | Wechsler Adult Intelligence Scale - Third Edition, Information subtest (Wechsler, 1997a) | Total score – Standard administration |
| MMSE | Mini-Mental State Examination (Folstein et al., 1975) | Total score – Standard administration |
| BDRS | Blessed Dementia Rating Scale (Blessed et al., 1998) | Total score – Standard administration |
| FAQ | Functional Activities Questionnaire (Pfeffer et al., 1992) | Total score – Standard administration |
| BDI | Beck Depression Inventory (Beck et al., 1961) | Total score – Standard administration |
| GDS | Geriatric Depression Scale (Sheikh and Yesavage, 1986) | Total score – Standard administration |
| *Cognitive tests* | | |
| PC-Vienna | PC-Vienna System (Schuhfried, 1992) | Cognitive reaction time (seconds), motor reaction time (seconds) – Standard administration |
| PASAT | Paced Auditory Serial Addition Test (Gronwall, 1977) | Correct responses – Calculation was removed to discard dependence on working memory. In contrast, participants compare numbers presented by saying if a given number is higher or lower than the preceding one |
| CTT | Color Trails Test (D’Elia, & Satz, 1989) | Part 1 (correct responses), Part 2 (correct responses), Part 1 minus Part 2 – Standard administration |
| Digit Span | Digit Span (Wechsler, 1997b) | Forward score, Backward score – Standard administration |
| Visuospatial Span | Visuospatial Span (Wechsler, 1997b) | Forward score, Backward score – Standard administration |
| Stroop | Stroop Test (Golden, 1978) | Sheet 1 (correct responses), sheet 2 (correct responses), sheet 3 (correct responses), interference index [Sheet 3 – (Sheet 1 * Sheet 2) / (Sheet 1 + Sheet 2)] – Standard administration |
| Verbal fluency | Verbal fluency (Benton et al., 1989; Piatt et al., 1999) | Letters (total words in three minutes), animals (total words in one minute), actions (total words in one minute) – Standard administration |
| Luria’s | Luria’s Premotor Functions (Christensen, 1979) | Luria’s hand alternative movements (number of correct movements), Luria’s motor coordination (number of correct movements), Luria’s motor inhibition (number of correct movements) – Standard administration |
| Logical Memory | Logical Memory (Wechsler, 1997b) | Immediate (correct responses), delayed (correct responses), recognition (correct responses) – Standard administration |
| TAVEC | *Test de Aprendizaje Verbal España-Complutense.* Spanish version of the California Verbal Learning Test (CVLT) | Learning (correct responses), delayed (correct responses), recognition (correct responses) – Standard administration |
| 8/30 SRT | In-house modification of 7/24 Spatial Recall Test (Rao et al., 1984). Previously described in Correia et al. (2015) | Immediate (correct responses), delayed (correct responses), recognition (correct responses) – Administration and scoring procedure was the same as described by Rao et al. (1984), but an eight-dot pattern was displayed on a 5x6 grid instead of the original one. Recognition consists of a forced choice trial between four similar options given two times |
| Visual Reproduction | Visual Reproduction (Wechsler, 1997b) | Immediate (correct responses), delayed (correct responses), recognition (correct responses) – Standard administration |
| Block Design | Block Design (Wechsler, 1997a) | WAIS-III total score, number of correct blocks in simple designs including 4 blocks, number of correct blocks in complex designs including 9 blocks, time (seconds) in the control task including 4 all-red blocks, time (seconds) in the control task including 9 all-red blocks – Besides standard administration, extended time to discard processing speed dependence was included. A control task was also given to assess manipulative ability (all-red blocks) |
| FRT | Facial Recognition Test (Benton et al., 1983) | Total score – Standard administration |
| JLOT | Judgment of Line Orientation Test (Benton et al., 1983) | Total score – Standard administration |
| TDAS | *Test de Denominación de Acciones y Sustantivos* (Nouns and Actions Naming Test). Previously described in Correia et al. (2015) | Nouns (correct responses and time, Actions (correct responses and time) – In-house computerized visual confrontation naming task including pictures of 40 nouns and 20 actions. Stimuli are presented and responses recorded with milliseconds precision using the E-prime v1.1 Software (Psychology Software Tools, Inc, 2002) |
| TGAAS | *Test de Generación de Acciones por Asociación Semántica* (Generating Actions by Semantic Association). Previously described in Ferreira et al. (2014) | Correct responses and time – In-house computerized auditory task where participants are given 30 nouns and semantic associated actions must be generated. Stimuli are presented and responses recorded with milliseconds precision using the E-prime v1.1 Software (Psychology Software Tools, Inc, 2002) |

**Supplementary Table 2. FreeSurfer methods for processing of T1-weighted MRI data**

| 1) Motion correction (Reuter et al., 2010). |
| --- |
| 2) Removal of non-brain tissue (Segonne et al., 2004). |
| 3) Automated Talairach transformation. |
| 4) Segmentation of the subcortical structures (Fischl et al., 2002; Fischl et al., 2004a). |
| 5) Intensity normalization (Sled et al., 1998). |
| 6) Tessellation of the gray matter white matter boundary. |
| 7) Automated topology correction (Fischl et al., 2001; Segonne et al., 2007). |
| 8) Surface deformation following intensity gradients to optimally place the gray/white and gray/cerebrospinal fluid borders at the location where the greatest shift in intensity defines the transition to the other tissue class (Dale et al., 1999; Dale and Sereno, 1993; Fischl and Dale, 2000). |
| 9) Surface inflation (Fischl et al., 1999a), registration to a spherical atlas (Fischl et al., 1999b). |
| 10) Parcellation of the cerebral cortex into units based on gyral and sulcal structure (Desikan et al., 2006; Fischl et al., 2004b). |
| 11) Creation of a variety of surface based data.  12) Smoothing of cortical data using a full-width half-maximum (FWHM) of 15 mm. |

References are provided at the end of the Supplementary Material.

**Supplementary Table 3. FSL methods for processing of DTI MRI data**

| **FDT (FMRIB’s Diffusion Toolbox)** |
| --- |
| 1) Correction of stretch and shear distortions produced by the gradient coils and correction of simple head motion using *eddycorrect*. |
| 2) Separation of the T2 sequence from diffusion directions using *fslroi*. |
| 3) Removal of non-brain tissue using BET (Smith, 2002). |
| 4) Extraction of Fractional Anisotropy (FA) and Mean Diffusivity (MD) maps with DTIFIT. |
| **TBSS (Tract-Based Spatial Statistics)** (Smith et al., 2006) |
| 1) Alignment of FA maps into a common space using the nonlinear registration tool FNIRT (Andersson 2007a, 2007b). |
| 2) Creation of a mean FA image and a mean FA skeleton that represents the centers of all tracts common to the study population. The matching between individual FA skeleton and the mean FA skeleton was carefully revised to confirm that tracts were properly aligned to the mean FA skeleton and the default 0.2 threshold was used. |
| 3) Application of original nonlinear registration to Mean Diffusivity (MD) data, and projection of each subject's aligned MD data onto the original mean FA skeleton, thereby allowing a voxel-wise cross-subject statistics. |

References are provided at the end of the Supplementary Material.

**References Supplemental Data**

Andersson JLR, Jenkinson M, Smith S. Non-linear optimisation. FMRIB technical report 2007a; TR07JA1, from www.fmrib.ox.ac.uk/analysis/techrep.

Andersson JLR, Jenkinson M, Smith S. Non-linear registration, aka Spatial normalisation. FMRIB technical report 2007b; TR07JA2, from www.fmrib.ox.ac.uk/analysis/techrep.

Beck A, Ward C, Mendelson M, Mock J, Erbaugh J. An inventory for measuring depression. Arch Gen Psychiatry 1961; 4: 561–71.

Benton A, Hamsher K, Sivan A. Multilingual aphasia examination. Iowa City, IA: AJA Associates (2nd Ed.). Iowa City: University of Iowa. 1989.

Benton A, Hamsher S, Varney O, Spreen N. Contributions to neuropsychological assessment: a clinical manual. New York: Oxford University Press. 1983.

Blessed G, Tomlinson B, Roth M. The association between quantitative measures of dementia and of senile change in the cerebral grey matter of elderly subjects. Br J Psychiatry 1968; 114: 797–811.

Christensen A. Luria's neuropsychological investigation (2nd Ed.). Copenhagen: Munksgaard. 1979.

Correia R, Nieto A, Ferreira D, Sabucedo M, Barroso J. Fund of information is more strongly associated with neuropsychological functioning than education in older Spanish adults. Arch Clin Neuropsychol 2015; 30: 310-21.

D’Elia L, Saltz P. Color Trail 1 and 2. Odessa, FL: Psychological Assesment Resources. 1989.

Dale AM, Fischl B, Sereno MI. Cortical Surface-Based Analysis I: Segmentation and Surface Reconstruction. Neuroimage 1999; 9: 179–94.

Dale AM, Sereno MI. Improved localization of cortical activity by combining EEG and MEG with MRI cortical surface reconstruction: a linear approach. J Cogn Neurosci 1993; 5: 162–76.

Desikan RS, Ségonne F, Fischl B, Quinn BT, Dickerson BC, Blacker D, Buckner RL, Dale AM, Maguire RP, Hyman BT, Albert MS, Killiany RJ. An automated labeling system for subdividing the human cerebral cortex on MRI scans into gyral based regions of interest. Neuroimage 2006; 31: 968–80.

Ferreira D, Molina Y, Machado A, Westman E, Wahlund L-O, Nieto A, Correia R, Junque C, Diaz-Flores L, Barroso J. Cognitive decline is mediated by gray matter changes during middle age. Neurobiol Aging 2014; 35: 1086-94.

Fischl B, Dale AM. Measuring the thickness of the human cerebral cortex from magnetic resonance images. Proc Natl Acad Sci U S A 2000; 97:11050–5.

Fischl B, Liu A, Dale AM. Automated manifold surgery: constructing geometrically accurate and topologically correct models of the human cerebral cortex. IEEE Trans Med Imaging 2001; 20:70-80.

Fischl B, Salat DH, Busa E, Albert M, Dieterich M, Haselgrove C, van der Kouwe A, Killiany R, Kennedy D, Klaveness S, Montillo A, Makris N, Rosen B, Dale AM. Whole brain segmentation: automated labeling of neuroanatomical structures in the human brain. Neuron 2002; 33: 341-55.

Fischl B, Salat DH, Van der Kouwe AJW, Makris N, Ségonne F, Quinn BT, Dale AM. Sequence-Independent Segmentation of Magnetic Resonance Images. Neuroimage 2004a; 23 Suppl 1: S69–S84.

Fischl B, Sereno MI, Dale AM. Cortical surface-based analysis. II: Inflation, flattening, and a surface-based coordinate system. Neuroimage 1999a; 9: 195-207.

Fischl B, Sereno MI, Tootell RB, Dale AM. High-resolution inter-subject averaging and a coordinate system for the cortical surface. Hum Brain Mapp 1999b; 8: 272–84.

Fischl B, van der Kouwe A, Destrieux C, Halgren E, Ségonne F, Salat DH, Busa E, Seidman LJ, Goldstein J, Kennedy D, Caviness V, Makris N, Rosen B, Dale AM. Automatically parcellating the human cerebral cortex. Cereb Cortex 2004b; 14: 11-22.

Folstein MF, Folstein SE, McHugh PR. Mini-mental- state. A practical method for grading the cognitive state of patients for the clinician. J Psychiatr Res 1975; 12: 189–98.

Golden C. Stroop Color and Word Test: A manual for clinical and experimental uses. Chicago: Stoelting. Illinois: Stoelting Company. 1978.

Gronwall D. Paced auditory serial-addition task: a measure of recovery from concussion. Percept Motor Skills 1977; 44: 367-73.

Pfeffer RI, Kurosaki TT, Harrah CH, Chance JM, Filos S. Measurement of functional activities in older adults in the community. J Gerontol 1982; 37: 323–9.

Piatt AL, Fields JA, Paolo AM, Tröster AI. Action (verb naming) fluency as an executive function measure: convergent and divergent evidence of validity. Neuropsychologia 1999; 37: 1499-503.

Rao SM, Hammeke TA, McQuillen MP, Khatri BO, Lloyd D. Memory disturbance in chronic progressive multiple sclerosis. Arch Neurol 1984; 41: 625-31.

Reuter M, Rosas HD, Fischl B. Highly Accurate Inverse Consistent Registration: A Robust Approach. Neuroimage 2010; 53: 1181–96.

Schuhfried G. Vienna Reaction Unit. Manual. Vienna: Schuhfried Ges.m.b.H. 1992.

Ségonne F, Dale AM, Busa E, Glessner M, Salat D, Hahn HK, Fischl B. A hybrid approach to the skull stripping problem in MRI. Neuroimage 2004; 22: 1060–75.

Ségonne F, Pacheco J, Fischl B. Geometrically Accurate Topology-Correction of Cortical Surfaces Using Nonseparating Loops. IEEE Trans Med Imaging 2007; 26: 518–29.

Sheikh J, Yesavage J: Geriatric Depression Scale (GDS): Recent evidence and development of a shorter version. Clin Gerontol 1986; 5: 165–73.

Sled JG, Zijdenbos AP, Evans AC. A nonparametric method for automatic correction of intensity nonuniformity in MRI data. IEEE Trans Med Imaging 1998; 17: 87–97.

Smith SM, Jenkinson M, Johansen-Berg H, Rueckert D, Nichols TE, Mackay CE, Watkins KE, Ciccarelli O, Cader MZ, Matthews PM, Behrens TE. Tract-based spatial statistics: Voxelwise analysis of multi-subject diffusion data. Neuroimage 2006; 31: 1487-505.

Smith SM. Fast robust automated brain extraction. Hum Brain Mapp 2002; 17: 143-55.

Wechsler D. Wechsler Adult Intelligence Scale - Administration and Scoring Manual, third ed. The Psychological Corporation, San Antonio, TX, 1997a.

Wechsler D. Wechsler Memory Scale - Third Edition. Technical Manual. San Antonio, TX: The Psychological Corporation. 1997b.
